# Genome reduction boosts heterologous gene expression in *Pseudomonas putida*

**DOI:** 10.1186/s12934-015-0207-7

**Published:** 2015-02-21

**Authors:** Sarah Lieder, Pablo I Nikel, Víctor de Lorenzo, Ralf Takors

**Affiliations:** Institute of Biochemical Engineering, University of Stuttgart, Allmandring 31, 70569 Stuttgart, Germany; Systems and Synthetic Biology Program, Centro Nacional de Biotecnología (CNB-CSIC), C/Darwin 3, 28049 Madrid, Spain

**Keywords:** *Pseudomonas putida*, Reduced genome, Heterologous gene expression, Chemostat, Energy maintenance, Metabolic engineering, Microbial cell factory

## Abstract

**Background:**

The implementation of novel platform organisms to be used as microbial cell factories in industrial applications is currently the subject of intense research. Ongoing efforts include the adoption of *Pseudomonas putida* KT2440 variants with a reduced genome as the functional *chassis* for biotechnological purposes. In these strains, dispensable functions removed include flagellar motility (1.1% of the genome) and a number of open reading frames expected to improve genotypic and phenotypic stability of the cells upon deletion (3.2% of the genome).

**Results:**

In this study, two previously constructed multiple-deletion *P. putida* strains were systematically evaluated as microbial cell factories for heterologous protein production and compared to the parental bacterium (strain KT2440) with regards to several industrially-relevant physiological traits. Energetic parameters were quantified at different controlled growth rates in continuous cultivations and both strains had a higher adenosine triphosphate content, increased adenylate energy charges, and diminished maintenance demands than the wild-type strain. Under all the conditions tested the mutants also grew faster, had enhanced biomass yields and showed higher viability, and displayed increased plasmid stability than the parental strain. In addition to small-scale shaken-flask cultivations, the performance of the genome-streamlined strains was evaluated in larger scale bioreactor batch cultivations taking a step towards industrial growth conditions. When the production of the green fluorescent protein (used as a model heterologous protein) was assessed in these cultures, the mutants reached a recombinant protein yield with respect to biomass up to 40% higher than that of *P. putida* KT2440.

**Conclusions:**

The two streamlined-genome derivatives of *P. putida* KT2440 outcompeted the parental strain in every industrially-relevant trait assessed, particularly under the working conditions of a bioreactor. Our results demonstrate that these genome-streamlined bacteria are not only robust microbial cell factories on their own, but also a promising foundation for further biotechnological applications.

**Electronic supplementary material:**

The online version of this article (doi:10.1186/s12934-015-0207-7) contains supplementary material, which is available to authorized users.

## Background

Much of contemporary metabolic engineering approaches, both at the laboratory scale and in industrial setups, mostly rely on the use of a few bacterial hosts as working platforms [1,2]. However, the organisms that are easiest to manipulate are often neither suitable nor entirely appropriate for specific large-scale and industrial applications. Several physiological and metabolic traits are desired in a robust production host [3-5]. In the first place, the platform cells must be hefty and able to endure a suite of environmental and process-related stresses [6]. Whenever possible, the cells should also exhibit decreased (and traceable) genetic drift, physically robust envelops, efficient and as-simple-as-possible transcription and translation controls, and predictable metabolic behavior [4]. Furthermore, the concept of a suitable host for biotechnological applications is reminiscent of that of a *minimal microbial cell*, in which all the elements deemed unnecessary for cellular functions other than replication and self-maintenance (e.g., prophages, flagellar genes, and cell-to-cell communication devices) have been eliminated. In spite of the evident need for a bacterial *chassis* reuniting all these desirable traits, only few hosts (typically *Escherichia coli* strains [7-11]) have been considered appropriate as biocatalysts in industrial endeavors, such as the production of functional recombinant proteins.

Building on the concepts outlined above, we advocate the choice of *Pseudomonas putida* strains as microbial platforms pre-endowed with metabolic and stress-endurance traits that are optimal for biotechnological needs [12]. In particular, the non-pathogenic *P. putida* strain KT2440 shows a remarkable metabolic diversity, amenability to genetic manipulation, and stress endurance, along with the welcome GRAS (*g*enerally *r*egarded *a*s *s*afe) status [13-16]. Sequencing of the 6,181,863-bp long genome of *P. putida* KT2440 brought forth a significant advance in the potential applications of this bacterium [17,18]. In an effort to enable the analysis of strain KT2440 from a systems biology perspective and to foster the development of its biotechnological applications, multiple tools for genome editing have been devised and implemented [19-22]. These tools have facilitated the design of a number of streamlined-genome (SG) variants derived from the wild-type strain. For instance, the construction and physiological characterization of a flagella-less variant of *P. putida* KT2440 (*P. putida* EM329) with some emergent properties, such an elevated NADPH/NADP^+^ redox ratio, was recently reported by Martínez-García et al. [23]. The physiological effects of freeing the bacterium of all the viral DNA encoded in its extant chromosome (represented by not less than four prophages) were likewise explored [24]. While such genetic manipulations conferred interesting biotechnological properties to the bacterial *chassis*, the industrial worth of a reduced genome *P. putida* strain has not been systematically explored hitherto. As a matter of fact, the rational engineering of cell factories tailored for optimized protein synthesis and process performance has traditionally focused on protein related issues (such as optimized codon usage, expression systems, folding characteristics, etc.), together with biochemical engineering aspects (i.e., bioreactor setup and control), while the basic properties of the biocatalyst proper are generally left to its default physiological values. However, expanding this scope by focusing on platform engineering opens the door to optimized strains offering low energy demands as a prerequisite for further improvements in production yield.

**Figure 1:**
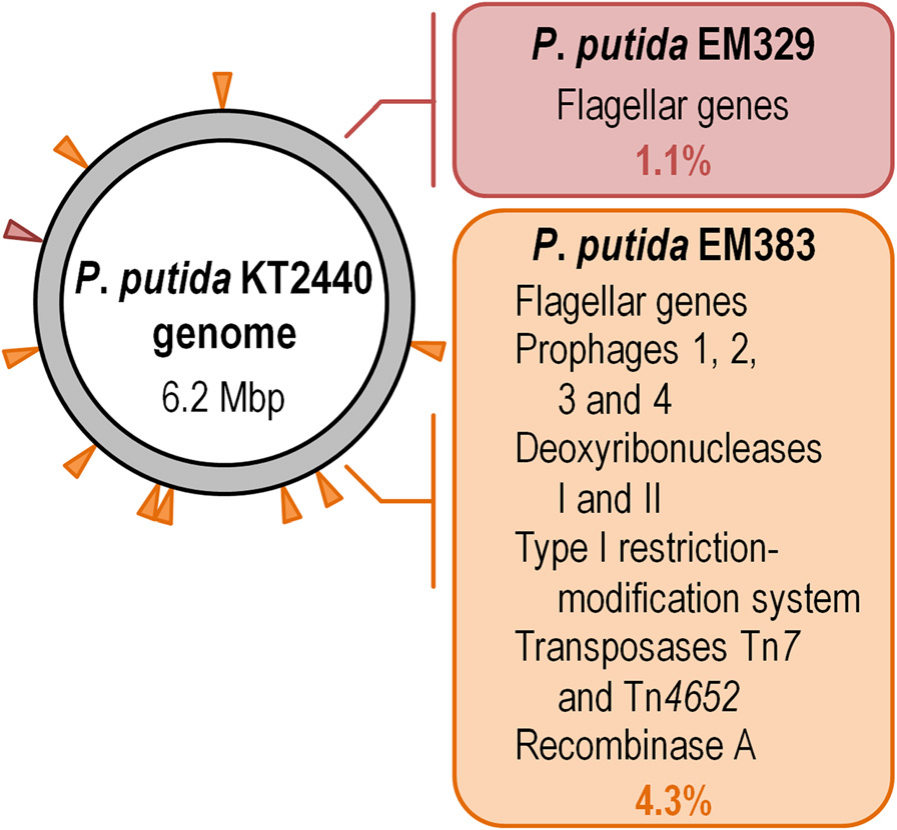
**Rationale behind the design of reduced-genome derivatives of** ***P*** **.** ***putida*** **KT2440.** Strains EM329 and EM383 were constructed using the seamless deletion system described by Martínez-García and de Lorenzo [20]. Note that while strain EM329 only lacks the genes encoding flagellar genes [23], the multiple deletions in strain EM383 were designed to endow the bacterium with the properties of a true microbial platform for a variety of applications. The relative physical location of the genes eliminated in the chromosome of *P. putida* KT2440 are indicated with slanted arrowheads and the percentage of the genome deleted is shown in each case. The red arrowhead represents the chromosomal location of the flagellar genes (deleted in strain EM329), while the orange arrowheads indicate the genes and gene clusters eliminated in strain EM383.

In this study, we have assessed the use of two heavily re-factored *P. putida* strains as potential hosts for protein production in a bioreactor setup. One of them lacks flagella, while the other carries multiple mutations implemented to ensure genetic and physiological stability (see Figure 1 and reference [25]). The well-known green fluorescent protein (GFP) from the jellyfish *Aequorea victoria* was selected as a model protein [26], and kinetic and physiological parameters related to cell performance were analyzed in both batch and continuous cultures. As shown below, the two re-factored versions of *P. putida* KT2440 outcompeted their parental strain in every parameter tested, showing an extraordinarily improved resistance to stress and enhanced protein production.

## Results and discussion

### Streamlined-genome *Pseudomonas putida* KT2440 as a *chassis* for heterologous protein production: design and construction of robust microbial cell factories

In this work, we have evaluated the properties of SG strains derived from *P. putida* KT2440 (Table 1) under the growth conditions and physiological regimes that prevail in an industrial setting. Figure 1 summarizes the genomic manipulations in each strain and the localization of the deleted elements in the chromosomal coordinates of *P. putida* KT2440. While the fundamental phenotypic changes brought about by deleting these genomic segments have been recently reported [23,25], this study deals with the question if the emergent properties previously detected in such strains can be exploited for improving heterologous protein production in a bioreactor.

**Table 1:** **Bacterial strains and plasmids used in this study**

**Bacterial strain or plasmid**	**Relevant characteristics** ^***a***^	**Source or reference**
*E. coli*		
DH5α	Cloning host; F^−^ λ^−^ *endA1 glnX44*(AS) *thiE1 recA1 relA1 spoT1 gyrA96*(Nal^R^) *rfbC1 deoR nupG* Φ80(*lacZ*Δ*M15*) Δ(*argF*-*lac*)*U169 hsdR17*(*r*_*K*_^−^ *m*_*K*_^*+*^)	[27]
*Pseudomonas putida*		
KT2440	Wild-type strain, spontaneous restriction-deficient derivative of strain mt-2 cured of the TOL plasmid pWW0	[28]
EM329	Flagella-less derivative of KT2440; ΔPP4329-PP4397 (flagellar operon)	[23]
EM383	Streamlined derivative of KT2440; ΔPP4329-PP4397 (flagellar operon) ΔPP3849-PP3920 (prophage 1) ΔPP3026-PP3066 (prophage 2) ΔPP2266-PP2297 (prophage 3) ΔPP1532-PP1586 (prophage 4) ΔTn*7* Δ*endA-1* Δ*endA-2* Δ*hsdRMS* Δflagellum ΔTn*4652*	[25]
Plasmid		
pSEVA234^*b*^	Expression vector; *oriV*(pBBR1) *lacI* ^*Q*^ *P*_*trc*_ *aphA*, Km^R^	[22]
pSEVA637^*b*^	Cloning vector carrying the green fluorescent protein gene; *oriV*(pBBR1) *aacC1*, Gm^R^	[22]
pS234G	Expression vector carrying the green fluorescent protein gene under control of the inducible P_*trc*_ promoter; *oriV*(pBBR1) *lacI* ^*Q*^ *P*_*trc*_→*gfp aphA*, Km^R^	This study

^*a*^Antibiotic markers: Gm, gentamicin; Km, kanamycin.

^*b*^Plasmids belonging to the SEVA (*S*tandard *E*uropean *V*ector *A*rchitecture) collection.

### Enhanced process parameters and energy profile of streamlined-genome derivatives of *Pseudomonas putida* KT2440 in continuous cultures

#### Biomass yield, carbon balances, and maintenance coefficients

**Figure 2:**
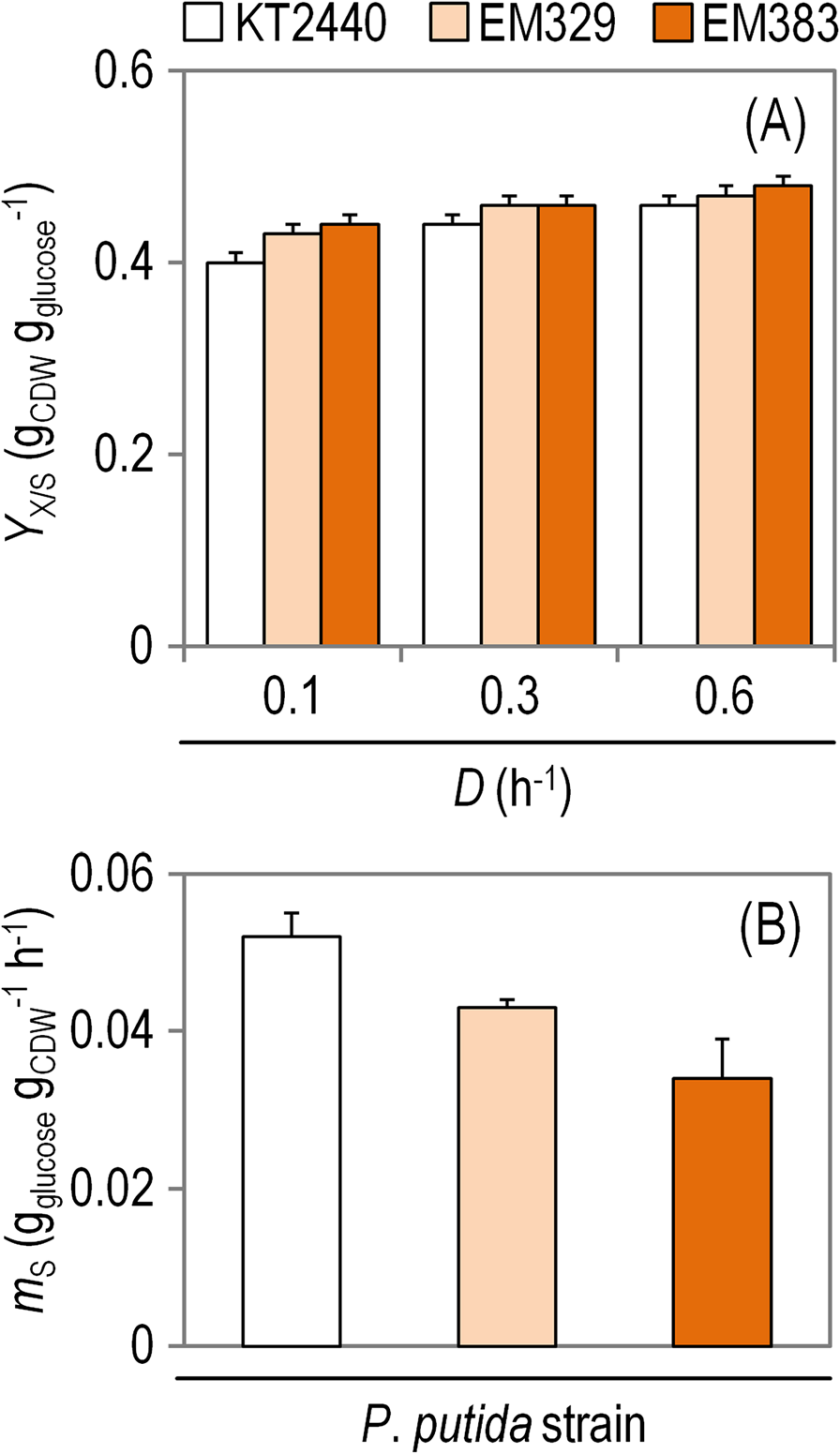
**Summary of the growth parameters for the different strains under study in glucose-limited chemostat cultures.** Shown are **(A)** the biomass yield coefficient (*Y*_X/S_), calculated at three different dilution rates *(D)*, and **(B)** the maintenance coefficient (*m*_S_). The growth parameters were calculated based on three independent biological experiments conducted in triplicate, and the bars represent the mean value of the corresponding parameter ± standard deviations.

The starting point in the characterization of the strains was the setup of continuous cultivations to explore the key kinetic and process parameters at different growth rates (Additional file 1: Figure S1). Yield coefficients, reflecting the efficiency in the conversion of the substrate into cell components, were calculated in glucose-limited continuous cultivations at steady-state conditions for various *D* values (Figure 2A). The mutant strains showed a higher *Y*_X/S_ value (statistically significant, *P* < 0.05) at all growth rates when compared to the wild-type strain. The highest difference (*ca*. 12%) was observed when comparing strain EM383 with wild-type KT2440 at *D* = 0.1 h^−1^. The differences between *P. putida* EM329 and EM383, on the contrary, were not statistically significant. The carbon emission rates (i.e., evolution of CO_2_) differed significantly between the strains. Averaging over all the tested *D* values, strains EM329 and EM383 had 9% and 16% lower CO_2_ formation, respectively, as compared to *P. putida* KT2440 (Additional file 1: Figure S2). This result suggests that the carbon substrate saved by-passing the synthesis of some cellular components (e.g., flagella) can be used for macromolecular biosynthesis, accompanied by a low evolution of CO_2_, an interesting trait for bioprocesses that depend on biomass formation. The next relevant question was whether these differences in biomass yields also correlate with energy maintenance.

The maintenance demand is an intrinsic characteristic of each specific microorganism. As it measures the amount of carbon source (and ATP) needed to maintain minimal functions within the cell other than generation of more biomass (i.e., non-growth processes), the lower the *m*_S_ value is for a given strain and/or culture condition, the higher the amount of carbon available to be used for ATP generation, NAD(P)H capacity, and also precursor availability. We explored this trait in the SG strains in the aforementioned glucose-limited continuous cultivations (Figure 2B). Maintenance was calculated *via* the specific rate of glucose uptake at different *D* values. The linear relationship between *q*_S_ and the respective growth rate was monitored over the range of *D* values comprised between 0.1 and 0.6 h^−1^. As *D* increased, so did the *q*_S_ values for each strain. By applying the Pirt's equation, an *m*_S_ of 0.052 ± 0.002 g_glucose_ g_CDW_^−1^ h^−1^ (corresponding to 0.29 mmol_glucose_ g_CDW_^−1^ h^−1^) was calculated for the wild-type *P. putida* strain. Note that no by-product formation needs to be taken into account for the strains considered, as *P. putida* does not produce any excretion metabolite under these conditions [29,30]. In fact, the carbon balances for all three strains showed an excellent closure (within the range 100% ± 2%) just by considering the formation of biomass, evolution of CO_2_, and the concentration of residual glucose in the culture medium (Additional file 1: Figure S2). The *m*_S_ values can also be transformed into ATP demands to directly visualize energy expenditures related to maintenance using some stoichiometric considerations. The Entner-Doudoroff pathway in *P. putida* yields 1 mole of ATP and 1 mole of NADH per mole of glucose consumed. Additionally, the tricarboxylic acid cycle forms 4 NADH and 1 FADH per each acetyl-coenzyme A, which, for the sake of simplicity in the calculations, can be lumped into 5 NADH. In consequence, 1 glucose molecule yields 1 ATP and about 11 NADH. Assuming a P/O ratio of 1.75 [15], 21 ATP per glucose are formed *via* oxidative phosphorylation. Under these assumptions, the *m*_ATP_ values (in mol_ATP_ g_CDW_^−1^ h^−1^) were 1.09 ± 0.06 for *P. putida* KT2440, and 0.91 ± 0.02 and 0.71 ± 0.05 for strains EM329 and EM383, respectively.

The *m*_S_ calculated from our experimental data is in the range of the *m*_S_ values reported by van Duuren et al. [31] for strain KT2440 in a similar chemostat setup. Vallon et al. [32] also found *m*_S_ values in the range of those reported here when studying a *P. putida*-based whole-cell biocatalysis process. The authors also pointed out that low *m*_S_ values seem to be typical for most *Pseudomonas* species. For the sake of comparison with a well established bacterial host used in industrial applications, the *m*_S_ calculated for *P. putida* KT2440 in this study was about 28% lower than that reported by Nanchen et al. [33] for wild-type *E. coli* MG1655 in a similar glucose-limited continuous culture. Interestingly, the two SG counterparts of *P. putida* KT2440 had lower *m*_S_ values than their parental strain. Specifically, strains EM329 and EM383 showed a reduction in their characteristic *m*_S_ values of 17% and 35%, respectively, when compared to the wild-type strain (*P* < 0.01). The corresponding *Y*_X/S_^*true*^ values were 0.47 g_CDW_ g_glucose_^−1^ for strain KT2440, and 0.49 g_CDW_ g_glucose_^−1^ for both EM329 and EM383. While the changes observed between the mutants and the wild-type strain were statistically significant, the difference when comparing the two SG variants was not.

According to the data available in the literature, maintenance coefficients of Gram-negative organisms grown in a defined glucose-containing medium vary from *ca*. 0.05 to 0.5 g_glucose_ g_CDW_^−1^ h^−1^ [34-37]. From this point of view, the calculated maintenance demands of *P. putida* and its SG derivatives lie within the lower end of the cited range of known *m*_S_ values. The emerging picture is that the deletion of cellular components and structures that spend energy (e.g., flagella assembly and motility) resulted in a reduction in maintenance demands in *P. putida*, rendering the SG strains appealing as production hosts.

### Energy status

**Figure 3:**
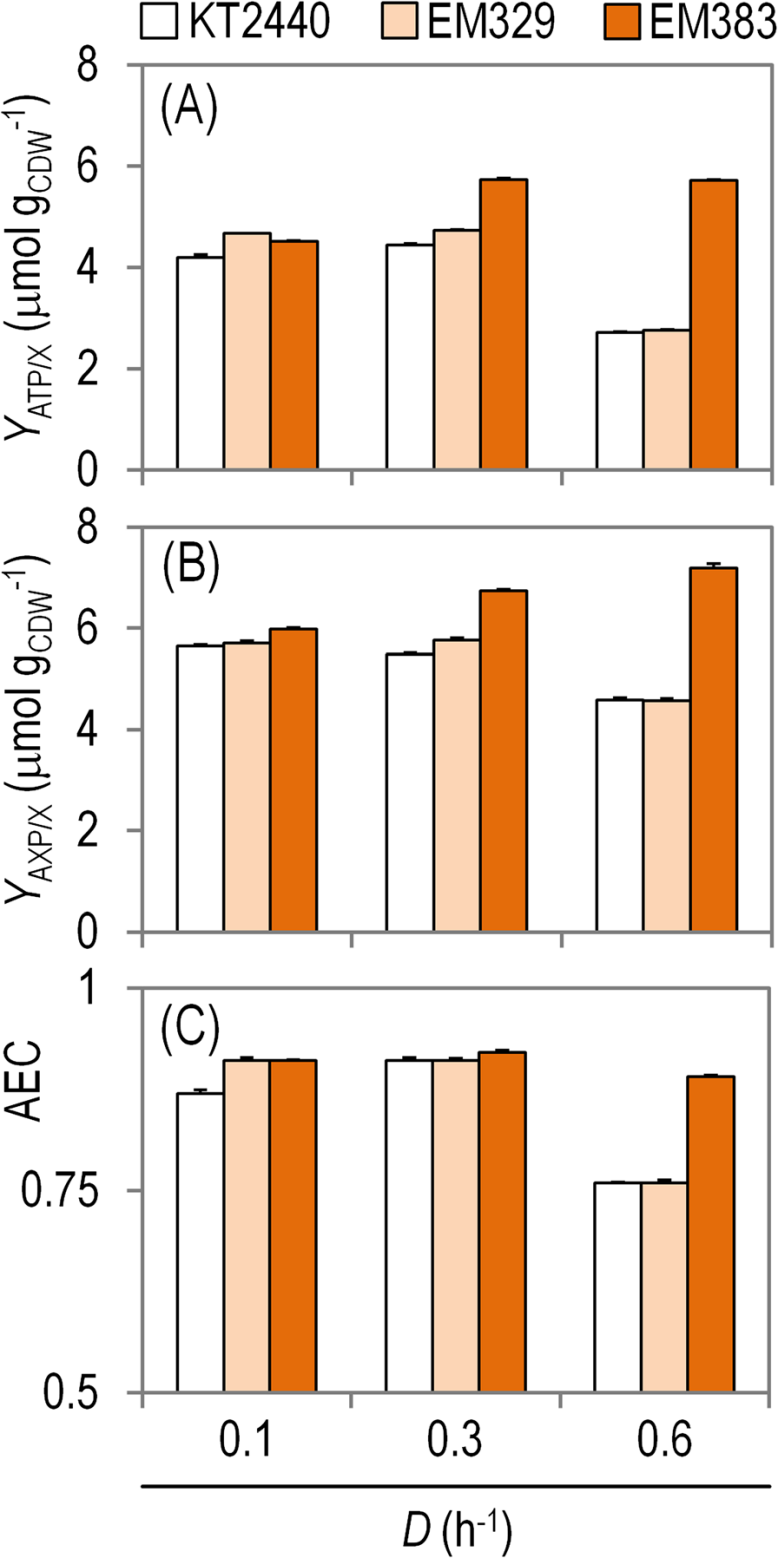
**Characterization of energy parameters for the different strains under study in glucose-limited chemostat cultures.** Shown are **(A)** the yield of ATP on biomass (*Y*_ATP/X_), **(B)** the yield of total nucleosides phosphates on biomass (*Y*_AXP/X_), and **(C)** the adenylate energy charge (AEC) of the cells at three different dilution rates **(D)**. The availability of phosphorylated adenine forms inside the cell and the AEC calculations are based on three independent biological experiments conducted in triplicate, and the bars represent the mean value of the corresponding parameter ± standard deviations.

During industrial production conditions, bacterial cells are constantly challenged with increased energy demands. The energetic capacity of the cells can be estimated *via* several physiological parameters, such as [i] the ATP/ADP ratio, [ii] the amount of ATP and the amount of total phosphorylated forms of adenine available per unit of biomass (*Y*_ATP/X_ and *Y*_AXP/X_, respectively), and [iii] the adenylate energy charge (AEC). The AEC gives a deeper insight into the energy state of the cells than the ATP/ADP ratio does, because it considers the relative contribution of all three phosphorylated forms of adenine. The energy capacity of the strains under study was explored in glucose-limited continuous cultivations at different *D* values (Figure 3). At all the *D* values tested, strain EM383 consistently revealed statistically significant higher ATP contents and AECs compared to both EM329 and KT2440 (*P* < 0.01) (Figure 3A and C). The total amount of the three possible phosphorylated forms of adenine was also high in the mutants, and particularly in strain EM383 at *D* = 0.6 h^−1^ (Figure 3B). Notably, under fast growth conditions, the difference in the ATP availability between strain EM383 with respect to both EM329 and KT2440 was more than doubled (Figure 3A). At the highest *D*, the AEC value dropped in all the strains, yet *P. putida* EM383 managed to keep a higher level of intracellular ATP even under these fast growth conditions, in contrast to the other two strains. These results are consistent with the decreased maintenances in the SG strains explained above, both at the level of substrate consumption and ATP availability.

Taken together, the results obtained in the glucose-limited continuous cultivations above suggested that both *P. putida* EM329 and EM383 have a number of physiological advantages over the wild-type KT2440 strain that could be potentially exploited for industrial purposes – such as expressing proteins encoded by heterologous DNA sequences. The systematic evaluation of these physiological traits on the background of heterologous protein production is explained in the next sections by adopting a model system that mimics industrial conditions.

### Evaluation of the streamlined-genome strains EM329 and EM383 as hosts for heterologous protein synthesis in batch cultures

Shaken-flask cultivation was selected as the first step in the characterization of the strains as microbial cell factories. Growth and physiological parameters as well as recombinant protein production were evaluated for each strain as explained below.

### Growth parameters and kinetics of GFP accumulation

**Figure 4:**
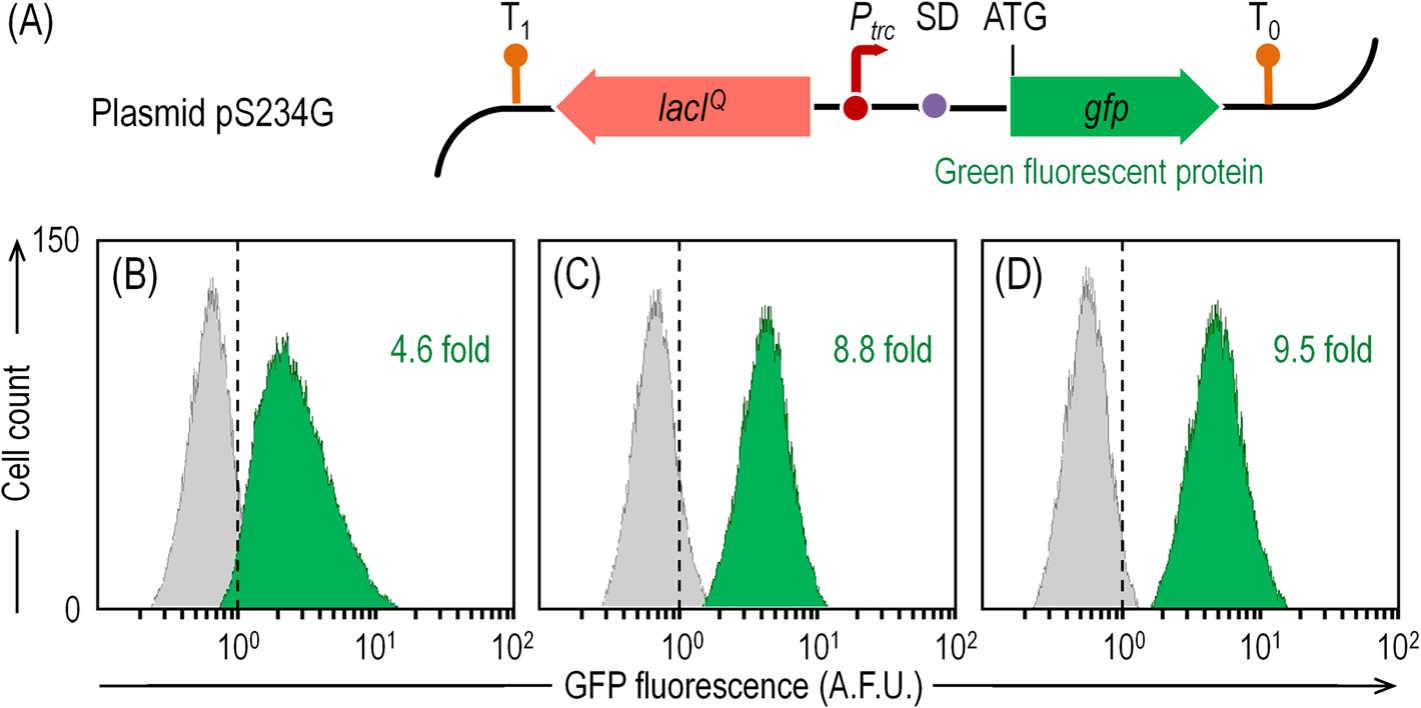
**Flow cytometry analysis of the green fluorescent protein accumulation in the strains under study. (A)** Schematic representation of plasmid pS234G, carrying *gfp* under the transcriptional control of the IPTG-inducible *P*_*trc*_ promoter. The activity of *P*_*trc*_ is controlled by the transcriptional regulator LacI^Q^. The transcriptional terminators included in the plasmid backbone are depicted as T_0_ and T_1_. The elements in this outline are not drawn to scale. *P. putida* KT2440 **(B)**, EM329 **(C)**, and EM383 **(D)** carrying pS234G were grown on M12 minimal medium containing glucose and harvested in mid-exponential phase. Gray and green peaks represent non-induced and induced cells, respectively. The vertical dashed line indicates the background fluorescence of the corresponding strain carrying the empty pSEVA234 plasmid, used as a negative control. The results shown are from a representative experiment, and the fold change in fluorescence upon induction is indicated in each case. A.F.U., arbitrary fluorescence units.

GFP was selected as the model protein to study heterologous protein synthesis in the different strains used in this study. A standardized version of *gfp*, derived from plasmid pSEVA637 [22], was cloned into a vector in which the gene transcription is under control of an IPTG-inducible expression system (i.e., a LacI^Q^/P_*trc*_ element). The resulting plasmid, termed pS234G (Figure 4A), was introduced in *P. putida* KT2440 and its SG derivatives, and their behavior was evaluated in shaken-flask cultures. The impact of introducing plasmid pS234G in these strains depended on the bacterial host, as both mutants had a lower reduction in their μ_max_ values than the wild-type did (Table 2). In strain KT2440, introduction of the *gfp*-expressing plasmid lowered μ_max_ in *ca*. 26% when compared to the plasmid-less counterpart. In the SG derivatives, this reduction never surpassed half that value (*ca*. 12%), demonstrating that the metabolic burden caused by plasmid maintenance and heterologous protein production had a low impact in strains EM329 and EM383. Both strains attained not only higher cell densities at the end of the 24-h cultivation period than KT2440, but they also grew faster irrespective of the plasmid they were transformed with. For instance, *P. putida* EM383/pS234G showed an 1.6-fold increase in μ_max_ with respect to KT2440/pS234G, and it also reached an 1.7-fold higher final CDW concentration.

**Table 2:** **Growth and protein synthesis parameters in shaken-flask cultures of different** ***P*** **.** ***putida*** **strains** ^***a***^

***P*** **.** ***putida***	**Plasmid** ^***b***^	**Growth parameters**		**Protein synthesis**	
		**μ**_max_^***c***^ **(h** ^−1^ **)**	**CDW** ^***d***^ **(g l** ^−1^ **)**	**π**_max_^***c***^ **(h** ^−1^ **)**	***Y***_GFP/X_^***c***^ **(A.F.U. g**_CDW_^−1^ **)**
KT2440	None	0.38 ± 0.01	2.6 ± 0.9	–	–
	pSEVA234	0.35 ± 0.02	2.1 ± 0.3	–	–
	pS234G	0.28 ± 0.03	1.7 ± 0.5	0.32 ± 0.06	2,125 ± 182
EM329	None	0.47 ± 0.02	2.9 ± 0.1	–	–
	pSEVA234	0.45 ± 0.01	2.9 ± 0.4	–	–
	pS234G	0.42 ± 0.04	2.7 ± 0.3	0.41 ± 0.02	2,613 ± 107
EM383	None	0.53 ± 0.01	3.4 ± 0.2	–	–
	pSEVA234	0.48 ± 0.02	3.1 ± 0.5	–	–
	pS234G	0.46 ± 0.03	2.9 ± 0.4	0.45 ± 0.01	3,047 ± 115

^*a*^Cells were grown batchwise in M12 minimal medium containing 10 g l^−1^ glucose as the sole carbon source and 1 mM IPTG was added in the cultures of the recombinant strains as indicated in Methods. Results represent the mean value of the corresponding parameter ± standard deviation of triplicate measurements from at least two independent biological replicates.

^*b*^Plasmid pS234G, a derivative of vector pSEVA234, carries *gfp* under control of an inducible LacI^Q^/P_*trc*_ element.

^*c*^Kinetic parameters were determined during exponential growth. μ_max_, maximum specific growth rate; π_max_, maximum specific rate of GFP formation; *Y*_GFP/X_, yield of GFP on biomass; A.F.U., arbitrary fluorescence units; −, not applicable.

^*d*^Final biomass concentration at 24 h. CDW, cell dry weight.

Another evident difference was that GFP had a better induction profile in strains EM329 and EM383 than in wild-type KT2440 (Figure 4). In fact, the difference between the induced *versus* the non-induced state in the mutants was twice as much as that observed in the parental *P. putida* strain (Figure 4B). The compactness of the Gaussian curves in flow cytometry experiments of both EM329 (Figure 4C) and EM383 (Figure 4D) also reflects a more homogenous induction of individual cells than in the wild-type strain, for which the curve in the cell counts *versus* GFP fluorescence plot was wider. When the trajectory of GFP formation was followed in batch cultures along the time, relevant differences were also observed (Table 2). Recombinant protein production is assumed to be proportional to growth. Accordingly, GFP accumulated faster in the SG strains (e.g., in *P. putida* EM383, π_max_ was 1.4-fold higher than in strain KT2440). For the calculation of GFP yield per biomass unit, the cell density of the culture was correlated to the emitted GFP fluorescence. A linear regression during the exponential growth phase resulted in a correlation factor of GFP fluorescence per unit of CDW, which allowed calculating the yield of recombinant protein (*Y*_GFP/X_). When biomass formation was taken into account to calculate the corresponding *Y*_GFP/X_ values, EM329 and EM383 also outcompeted *P. putida* KT2440 in 20% and 39%, respectively.

### Enhanced cell viability of the streamlined-genome strains expressing *gfp*

The slight decrease in μ_max_ and in the final cell density of the recombinant strains expressing *gfp* (Table 2) suggested that the metabolic burden imposed by protein accumulation could affect final yields and the overall process performance. On this background, we wondered whether cell viability could be affected as well [38], and we resorted to the PI exclusion test to explore this possibility (Additional file 1: Figure S3). While *P. putida* KT2440 showed a decrease in cell viability in the presence of pS234G as compared to the same strain with an empty plasmid, neither EM329 nor EM383 showed differences in the PI staining profile. Moreover, the percentage of PI-stained cells was lower for both SG strains than for the parental host, irrespective of the plasmid they carried. Among the strains tested, *P. putida* EM383 showed the highest cell viability. Notably, when the strains bearing plasmids were compared with their plasmid-free counterparts, no decrease in cell viability was observed in strains EM329 and EM383 (data not shown). When the same comparison was established for KT2440, a significant increase (*ca*. 25%) of the PI-positive population was detected in the strains carrying plasmid DNA as compared to the plasmid-free host, a figure in agreement with the results of Table 2. These results suggest that the SG *P. putida* strains have not only a high ability of carrying and replicating heterologous plasmid DNA (see below), but also that they tolerate the metabolic burden commonly associated with plasmid replication better than wild-type *P. putida* KT2440.

### Plasmid stability

All the recombinant cells were able to maintain the recombinant plasmid after 24 h of cultivation, with no significant differences among the three strains. However, when the percentage of plasmid-bearing cells was estimated after 48 h of cultivation, a significant difference in the segregational stability of pS234G could be observed. While *P. putida* KT2440 and EM329 cells retained the plasmid up to 81% ± 1% and 85% ± 4% of the total bacterial population, strain EM383 had a percentage of recombinants that reached 100% ± 2% (*P* < 0.05, when compared to the other two strains). In other words, strain EM383 did not show any significant plasmid loss after prolonged cultivation, reflecting a higher stability of extra-chromosomal DNA. This phenomenon is consistent with the absence of some recombinogenic features in this strain (e.g., Tn*7* and Tn*4652* transposases) that are known to bring forth genetic instability [39,40]. Deletion of these elements results in significant genome and plasmid stabilization, which in turns is beneficial in industrial processes with long fermentation runs [38,41].

### Kinetics of GFP formation in bioreactor batch cultures: influence of controlled aeration and carbon source on growth and profile of protein synthesis

By judging the process parameters measured in shaken-flask cultures, the viability profile of the recombinants under these conditions, and the genetic stability of the cells, we concluded that the SG strains were advantageous bacterial hosts for protein synthesis in comparison with wild-type KT2440. Building on this hypothesis, we further evaluated their capabilities as microbial cell factories in the well-controlled environment of a bioreactor under conditions compatible with industrial production.

### Growth parameters

**Figure 5:**
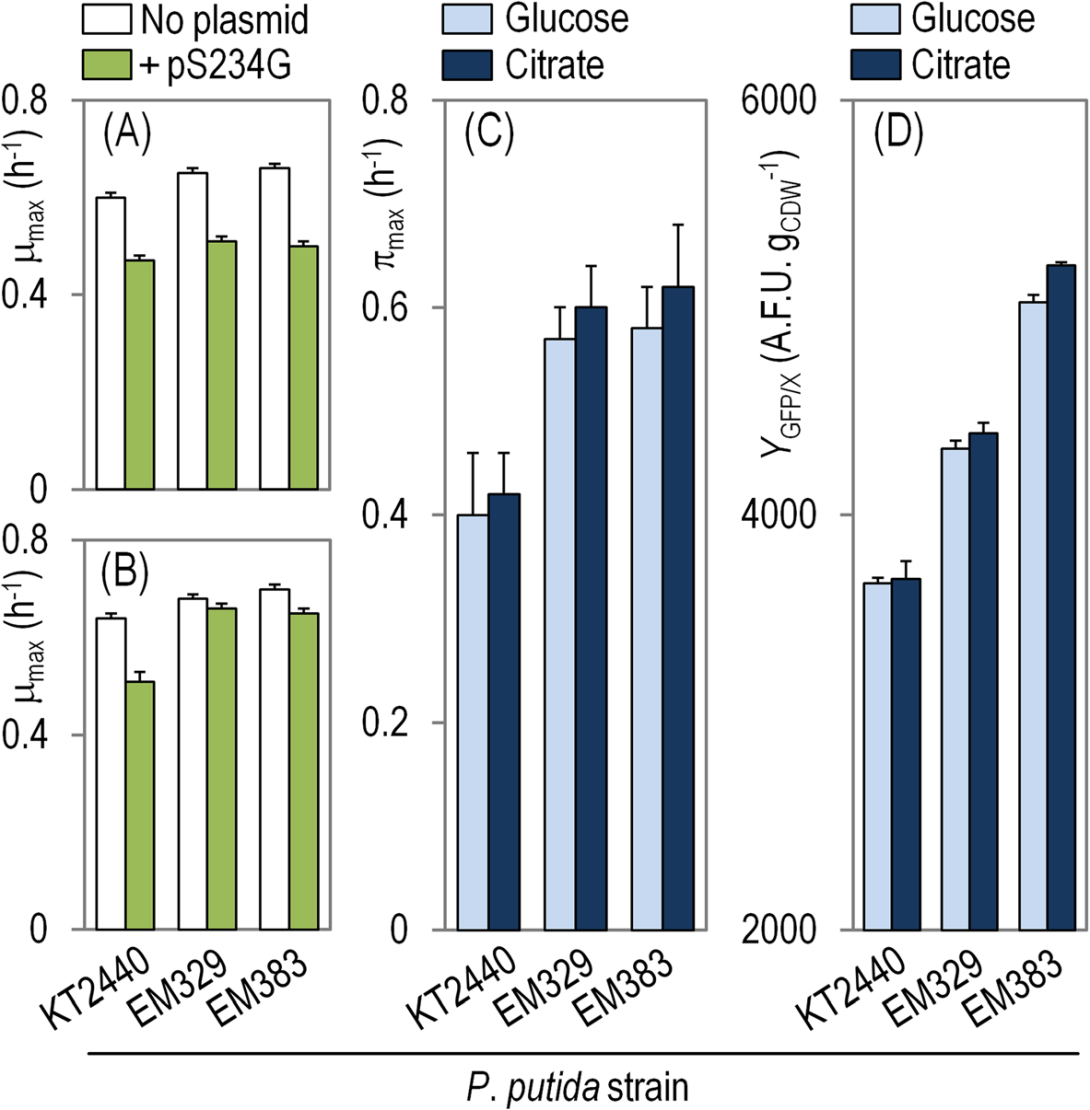
**Growth parameters and protein production kinetics for the strains under study in batch bioreactor cultures.** Shown are the specific growth rate (μ_max_) for cells grown on **(A)** glucose and **(B)** citrate, as well as the effect of plasmid maintenance and heterologous protein production under these growth conditions. The accumulation of the green fluorescent protein (GFP) in cultures of the strains carrying pS234G was assessed during exponential growth on M12 minimal medium containing either glucose or citrate through **(C)** the maximum specific rate of GFP formation (π_max_) and **(D)** the yield of GFP on biomass (*Y*_GFP/X_). The growth parameters and protein production kinetics were calculated based on three independent biological experiments conducted in triplicate, and the bars represent the mean value of the corresponding parameter ± standard deviations.

The derivative SG strains reached statistically significant higher μ_max_ values than the wild-type KT2440 strain in all the cultivations performed (Figure 5). When grown on glucose as the sole carbon source, EM329 showed a 7% and EM383 a 10% increase in μ_max_ (Figure 5A, *P* < 0.05). When using citrate as the carbon source, EM329 showed 4% and EM383 showed 11% faster growth (Figure 5B, *P* < 0.05). Mutant EM383 also reached statistically significant higher μ_max_ compared to strain EM329. Besides, both EM329 and EM383 attained higher final CDW concentrations when grown on glucose as the sole carbon source (9% and 13%, respectively when compared to *P. putida* KT2440; *P* < 0.05) (Additional file 1: Figure S4), mirroring the results already observed in shaken-flask cultures (Table 2). This difference was not observed on citrate, as all the strains reached a similar final biomass density (Additional file 1: Figure S5). In all, these results show the importance of adequate aeration and mixing within the bioreactor. In the first place, all the strains attained higher μ_max_ values and final cell densities in bioreactor cultivations as compared to the same traits in shaken-flask cultures. On the other hand, as both *P. putida* EM329 and EM383 are devoid of the flagellar machinery that would enable the cells to explore different microenvironments within the bioreactor, they tend to sediment and, if not properly stirred, the cells will likely become limited in O_2_ transfer, as previously hinted by Martínez-García et al. [23]. The same stirring speed and air bubbling applied to the bioreactor to grow *P. putida* KT2440 enabled a much better growth profile of the SG strains.

In a subsequent step, the influence of GFP production on the growth of the SG strains was investigated. As a further control, the wild-type strain was also transformed with the empty vector pSEVA234. Introduction of the empty vector in KT2440 did not result in a significant decrease in growth (1.5% in average), as it was also quantified in shaken-flask cultures (Table 2). On the basis of these results, the influence of the control vector on the physiology of the cells was deemed negligible. Expression of *gfp* from plasmid pS234G, on the contrary, caused an average 6% decrease in the μ_max_ value for the wild-type strain. On the other hand, expression of *gfp* in the SG strains did not lead to any significant decrease of μ_max_. The general trend of an increase in μ_max_ for the derivative strains previously observed in all the growth conditions analyzed could also be observed under recombinant protein expression conditions, particularly when using citrate as the sole carbon source. In fact, when growing on citrate, *P. putida* EM329/pS234G and EM383/pS234G reached significantly higher growth rates (*ca*. 32% for both strains) than *P. putida* KT2440/pS234G (Figure 5A and B, *P* < 0.05). No significant differences, however, were observed within the two derivative strains, as they grew very similarly and attained very comparable final cell densities.

### Recombinant protein expression

During exponential growth of the cells, the trajectory of fluorescence increase due to GFP accumulation was found to be exponential as well (Additional file 1: Figure S4 and S5). Under these production conditions, the π_max_ values were higher in the reduced genome strains as compared to the wild-type, in an almost carbon source-independent fashion (Figure 5C). The highest differences were detected using citrate as the carbon source; under these conditions, *P. putida* EM329/pS234G and EM383/pS234G showed an increase of 43% and 48% in π_max_, respectively, when compared to the same parameter in *P. putida* KT2440/pS234G (*P* < 0.05). Both derivative strains also had a significantly higher *Y*_GFP/X_ compared to the wild-type strain (Figure 5D, *P* < 0.05), and this trend was again more or less independent of the carbon source used. For instance, when growing the cells on glucose, EM329 reached a 18% higher yield than the wild-type strain, whereas EM383 was capable of attaining a 37% higher yield than strain KT2440. On citrate, the differences between the *Y*_GFP/X_ values for strains EM329 and EM383 were 20% and 41%, respectively, as compared to wild-type KT2440. In the exponential growth phase, the volumetric productivity of GFP was estimated to be 3,470 ± 9 A.F.U. l^−1^ h^−1^ for strain EM383/pS234G when growing on citrate, the highest among the strains and growth conditions tested in this study.

### Organic acids formation

One important aspect of industrial fermentations is the spillage of by-products that divert carbon (and, most often, also cofactors such as ATP or NADPH) needed for the synthesis of the desired product [42]. As mentioned before, *P. putida* does not secrete metabolites at a high concentration, as it is the case, for example, of acetate in *E. coli* fermentations [43]. However, when glucose is used as the carbon source, part of the substrate is usually oxidized by *P. putida* to gluconate in the cell periplasm by the activity of a glucose dehydrogenase [30]. Gluconate can leak out of the cell into the culture medium and is re-used as substrate as growth proceeds. When the accumulation of gluconate in the culture medium was evaluated in the bioreactor cultures, a sharp peak for *P. putida* KT2440 was observed in mid-exponential growth phase, reaching 18.5 ± 3.1 mM (i.e., *ca*. 3.5 g l^−1^). In contrast, both SG derivatives produced less gluconate, its concentration reaching 10.2 ± 1.4 and 9.3 ± 1.5 mM, respectively. These figures are comparable to those obtained when gluconate formation was evaluated in cultures of the strains carrying plasmid pS234G (data not shown). The kinetics of gluconate accumulation was very similar among all the strains, and this metabolite altogether disappeared from the culture supernatants in the late exponential phase as it was likely used by the cells as substrate. As a consequence of this significant reduction in the oxidation of glucose in the mutants, it is likely that more carbon is readily available for catabolism, in agreement with the high *Y*_X/S_ values and CDW concentrations observed in the cultures of both *P. putida* EM329 and EM383.

## Conclusion

Determinants of successful recombinant protein production, such as the rate and duration of production and quality or stability of the product, strongly depend on the physiology of the producer cell [44]. These traits can be typically manipulated by metabolic engineering of the host cell, by genetic engineering of the expression vector, and also by means of process engineering [45,46]. However, the vast majority of metabolic engineering efforts so far have dealt with the manipulation of genetic parts implanted in a bacterial host, optimization of the process parameters, and less so with the microbial *chassis* itself.

In the case of the environmental bacterium *P. putida*, the set of genes strictly needed for survival in soil is most likely not the gene complement appropriate for the efficient production of heterologous proteins in an industrial setup. In particular, the deletion of the flagellar operon clearly resulted in a physiological advantage in our experimental setup, in which motility is not a required feature – and it is even a detrimental one, as it consumes large amounts of ATP [47,48]. The elimination of flagella in *P. putida* KT2440 not only causes a surplus of ATP but also of NADPH [23] that can be potentially funneled into heterologous pathways. Moreover, elimination of the proviral DNA in strain KT2440 makes cells more resistant to DNA insults [24], such as UV radiation, along with a moderate improvement of ATP and NAD(P)H levels [25]. We thus anticipate that there is an additive physiological effect of the two types of deletions along with an increase of resistance to DNA damage. Further benefits can be brought forth by the removal of the other genomic DNA segments of *P. putida* that were erased in strain EM383. The separate contribution of every deletion was not addressed, as it is possible that the breakdown is not identical for each heterologous gene or pathway that can be expressed in the SG strains. In this work, we have instead focused on the positive effect of merging all deletions for the sake of designing a bacterial *chassis* useful for the industrial production of heterologous proteins.

Genome reduction of the wild-type *E. coli* strain MG1655 by Blattner and collaborators resulted in advantages (mostly in terms of genetic stability) for hosting and expressing heterologous genes [49-52]. While significant reductions of the *E. coli* MG1655 genome size have been achieved thus far [10], these strains retain the genomic and biochemical frame of a typical enterobacterium. This is a significant issue for expression of recombinant genes or pathways that cause stress or demand a high ATP and/or NAD(P)H availability to achieve full functionality [53,54], as it is the case with the *P. putida* variants examined in this article. In this context, the results presented above showed that the two SG derivatives of *P. putida* KT2440 outcompeted the parental strain in every biotechnologically-relevant parameter assessed among all the culture conditions tested, particularly in a bioreactor setup.

As shown above, *P. putida* EM329 and EM383 are not only sound microbial cell factories on their own, but they also provide a solid foundation for further targeted manipulations of their genomes. These forthcoming operations will not only result in enhanced bacterial *chassis* tailored for industrial protein synthesis, but they will also shed light on the relevant question about what is the minimal set of genes needed to maintain cell functioning, fitness, and robustness. Moreover, the combination of these genomic surgery strategies along with the optimization of industrial cultivation parameters (e.g., by analyzing protein production in fed-batch cultures) will certainly result in significant improvements of the overall process performance in a variety of biotechnological applications.

## Methods

### Bacterial strains, culture media, and general procedures

Bacterial strains and plasmids used in this study are listed in Table 1. *E. coli* and *Pseudomonas* strains were routinely grown at 37°C and 30°C, respectively, in rich LB medium [55] under oxic conditions (i.e., in Erlenmeyer flasks containing medium up to one-tenth of their nominal volume with agitation at 170 r.p.m.). *E. coli* DH5α was used for routine cloning procedures and plasmid maintenance. The physiological characterization of *P. putida* recombinants was carried out both in shaken-flask and bioreactor cultures using M12 minimal medium, which contained 2.2 g l^−1^ (NH_4_)_2_SO_4_, 0.4 g l^−1^ MgSO_4_ · 7 H_2_O, 0.04 g l^−1^ CaCl_2_ · 2H_2_O, 0.02 g l^−1^ NaCl, 2 g l^−1^ KH_2_PO_4_, added with trace elements (2 mg l^−1^ ZnSO_4_ · 7H_2_O, 1 mg l^−1^ MnCl_2_ · 4H_2_O, 15 mg l^−1^ Na_3_citrate · 2H_2_O, 1 mg l^−1^ CuSO_4_ · 5H_2_O, 0.02 mg l^−1^ NiCl_2_ · 6H_2_O, 0.03 mg l^−1^ Na_2_MoO_4_ · 2H_2_O, 0.3 mg l^−1^ H_3_BO_3_, and 10 mg l^−1^ FeSO_4_ · 7H_2_O). All cultivations were started using cells from a single colony in an LB medium plate, growth and harvested from exponential phase cultures in LB medium, and stored as a working cryo-culture bank at −70°C as a 20% (v/v) glycerol stock. Glucose or citrate were used as representative glycolytic or gluconeogenic carbon sources, respectively, throughout this study. The concentration of each carbon source in pre-cultures was 4 g l^−1^, while in batch cultivations (both in shaken-flasks and bioreactors) it was increased up to 10 g l^−1^. All solid media used in this work contained 15 g l^−1^ agar, and, whenever needed, kanamycin was added at 50 μg ml^−1^ as a filter-sterilized solution for plasmid maintenance. Isopropyl-β-D-thiogalactopyranoside (IPTG) was added at 1 mM to induce the expression of genes under the control of LacI^Q^/*P*_*trc*_. Growth was estimated in an Ultrospec 3000 *pro* UV/Visible spectrophotometer (GE Healthcare Bio-Sciences Corp., Piscataway, NJ, USA) by measuring the optical density at 600 nm (OD_600_) after diluting the culture as necessary with 9 g l^−1^ NaCl. In bioreactor cultivations, the cell dry weight (CDW) was measured in culture aliquots as appropriate for further mass-based calculations. CDW was determined in 10-ml culture samples by transferring the broth into previously-weighed glass tubes. The suspension was centrifuged at 7,000 r.p.m. and 4°C for 10 min and washed twice with 5 ml of cold saline. The pellet fraction was finally dried at 85°C until constant weight (*ca*. 48 h). The yield of biomass on substrate (*Y*_X/S_, in g_CDW_ g_glucose_^−1^) was derived from the CDW assessed in the samples and the glucose consumption rates (see below).

### Bioreactor cultures

All bioreactor cultures were carried out in an *in-situ* sterilizable bench-top 3.7 liter fermentor (Bioengineering AG, Wald, Switzerland). Exhaust gas composition (CO_2_ and O_2_), dissolved O_2_ concentration, and pH in the liquid phase were monitored on line using BCP-CO_2_ and BCP-O_2_ analyzers (BlueSens GmbH, Herten, Germany) and O_2_ and pH probes (Mettler Toledo GmbH, Giessen, Germany). The measurement of CO_2_ in the exhaust gas was used to calculate CO_2_ emission rates. The dissolved O_2_ concentration was monitored to assure non-limiting oxic conditions. In all cultivations, the dissolved O_2_ level was kept higher than pO_2_ = 70% (pO_2_ = 100% is defined as the dissolved O_2_ level in the bioreactor under operating conditions, but without biomass in suspension). A pre-culture was prepared for each run by inoculating cells from a working cryo-culture bank (8.5 ml) in 150 ml of M12 minimal medium contained in a 1.5-liter baffled Erlenmeyer flask. Cells were cultivated as explained above until the culture reached OD_600_ = 1.5 and used as the inoculum as explained below.

### Batch cultivation

Bioreactor batch cultivations were inoculated aseptically with a mid-exponential, shaken-flask pre-culture to reach a final working volume of 1.5 liters. Previous to inoculating the bioreactor, the operating conditions were set to 30°C, a stirrer speed of 700 r.p.m., an over-pressure in the vessel of 0.5 bar, and an aeration of 2 l min^−1^ of filtered-sterilized ambient air. The pH was set and maintained at pH = 7.0 by automatic addition of 25% (v/v) NH_4_OH.

### Continuous cultivation

In the case of glucose-limited continuous cultivations, the batch cultivation was switched into chemostat operation when glucose was completely depleted. The dilution rate (*D*) was increased stepwise from *D* = 0.1 h^−1^ to 0.3 h^−1^, and finally to 0.6 h^−1^. Each *D* value, determined by feeding medium at a pre-defined flow rate, was maintained for 5 residence times under steady-state conditions before further increasing the growth rate. The weight gain of the bioreactor was constantly monitored, and a harvest pump was started whenever the weight gain exceeded 10 g. Additionally, *D* values were manually checked by weighing the mass of the harvest outflow within a time-span of 1 h before sampling.

### Nucleic acid manipulation, plasmid construction, and plasmid stability assay

DNA manipulations followed well established protocols [55]. Plasmid pS234G carries the green fluorescent protein gene under transcriptional control of the IPTG-inducible P_*trc*_ promoter. This expression vector was constructed as follows. Plasmid pSEVA637 was digested with *Hin*dIII and *Spe*I, and the *ca*. 0.7-kb DNA fragment, spanning *gfp* preceded by a synthetic ribosome binding site, was ligated into pSEVA234 restricted with the same enzymes. The ligation mixture was transformed in *E. coli* DH5α, and positive clones were identified in LB medium plates containing kanamycin. Plasmid DNA was recovered from single clones and checked by automated sequencing. Plasmids were transferred into *P. putida* KT2440 and its derivatives by electroporation [56].

Plasmid segregational stability in cells grown in shaken-flask cultures was estimated as described by Nikel and de Lorenzo [57]. Briefly, cultures were serially diluted in 10-fold steps in LB medium containing no antibiotics. The dilution level was estimated based on OD_600_ measurements of the samples, and 50 μl of the final dilution was plated onto LB medium plates with and without kanamycin. Colony forming units (CFUs) were counted after 24 h of growth at 30°C in biological triplicates. The segregational stability of pSEVA234 and pS234G was calculated by comparing CFUs in plates with and without kanamycin.

### Analytical procedures

#### GFP quantification

##### Determination of GFP fluorescence by flow cytometry

Cells sampled from shaken-flask cultures at the time points indicated in the text were immediately diluted with phosphate-buffered saline to an OD_600_ of *ca*. 0.35 and fixed with 0.4% (v/v) formaldehyde. Flow cytometric analysis of GFP fluorescence levels was performed in a Gallios™ flow cytometer (Beckman Coulter Inc., Indianapolis, IN, USA) equipped with an argon ion laser of 15 mW at 488 nm as the excitation source. Size-related forward scatter signals gathered by the cytometer were analyzed using the Cyflogic™ 1.2.1 software (CyFlo Ltd., Turku, Finland) to gate fluorescence data only from bacteria in the stream. The green fluorescence emission was detected using a 530/30-nm band pass filter set. Data for >15,000 cells per experiment were collected, and the Cyflogic™ 1.2.1 software was used to calculate the geometric mean of fluorescence per bacterial cell (*x*-mean) in each sample.

##### Determination of GFP fluorescence by spectrofluorimetry

Fluorescence in samples from bioreactor cultures was determined by taking 200-μl technical triplicates of the cell suspension and the corresponding filtrates into a 96-well microtiter plate. The fluorescence was quantified after 60 min (allowing GFP to mature) at 485 nm (excitation) and 535 nm (emission) in a fluorescence microplate analyzer (Synergy 2, BioTek Instruments, Inc., Winooski, VT, USA). The yield of GFP on biomass [*Y*_GFP/X_, in arbitrary fluorescence units (A.F.U.) g_CDW_^−1^] was derived from these measurements.

##### Kinetics of GFP accumulation in bioreactor cultures

The trajectory of GFP increase was analyzed throughout the growth curve in batch cultures. A factor, termed π_max_, was implemented to describe the increase of GFP over time. This factor is analogous to μ, the specific growth rate, which describes the increase of biomass over time during exponential growth. The corresponding equation is:

$$ {C}_{\mathrm{P}}={C_{\mathrm{P}}}^0\times \exp \left({\uppi}_{\max}\times t\right) $$

where *C*_P_ is the GFP fluorescence (in A.F.U.), *C*_P_^0^ is the GFP fluorescence at *t* = 0 h (in A.F.U.), π_max_ is the maximum specific rate of GFP formation (in h^−1^), and *t* is time (in h).

##### Cell viability

We resorted to the propidium iodide (PI, a strong DNA intercalating agent) test, based on dye exclusion, to estimate the cell viability in samples from shaken-flask cultures. Cells having intact, polarized membranes are able to interact with and to exclude charged molecules like PI, while dead or seriously damaged bacteria become stained with the dye [58]. Flow cytometry analysis was performed to evaluate the percentage of PI-stained cells as a measure of cell viability. Measurements were performed in a Gallios™ flow cytometer (Beckman Coulter Inc.), using the argon ion laser at 488 nm as the excitation source. The characteristic PI fluorescence emission at 617 nm was detected using a 620/30-nm band pass filter array. PI (Life Technologies Corp., Grand Island, NY, USA) was used from a freshly-prepared stock solution at 0.5 mg ml^−1^ in water and added to a final concentration of 1.5 μg ml^−1^ to the cell suspension. Cells were stained for 30 min in the dark, and measured thereafter.

##### Quantification of glucose and organic acids

The concentration of residual glucose and citrate in the supernatants was quantified using commercial kits according to the manufacturer's instructions (R-Biopharm AG, Darmstadt, Germany). The evolution of gluconate was also followed using a similar procedure, using a kit from Megazyme International Ireland (Bray, Ireland). In either case, control mock assays were conducted by spiking M12 minimal medium with different amounts of the carbon source under examination.

##### Determination of ATP/ADP ratios, ATP yields, and the adenylate energy charge

Biocatalytic reactions in the cells were stopped by promptly mixing the samples with 35% (w/v) HClO_4_. A 4-ml sample was taken with a fast sampling probe directly into 1 ml of pre-cooled (−20°C) HClO_4_ solution on ice and mixed immediately. The sample was shaken at 4°C for 15 min in an overhead rotation shaker. Afterwards, the solution was neutralized on ice by fast addition of 1 ml of 1 M K_2_HPO_4_ and 0.9 ml of 5 M KOH. The neutralized solution was centrifuged at 4°C and 22,000 r.p.m. for 10 min to remove cell debris, and precipitated proteins and KClO_4_. The supernatant was kept at −20°C for batch high pressure liquid chromatography (HPLC) measurements. At each sampling time, a broth sample containing cells and a filtrated sample without cells was treated according to this procedure. Nucleotide analysis was performed by reversed-phase ion-pair HPLC. The HPLC system (Agilent Technologies GmbH, Waldbronn, Germany) consisted of an Agilent 1200 series auto-sampler, binary pump, thermostated column compartment, and a diode array detector set at 260 and 340 nm. The nucleotides were separated and quantified on a reversed-phase C18 column combined with a security guard column (Supelcosil LC-18-T, 25 cm × 4.6 mm, 3 μm particle size, equipped with 2-cm Supelguard LC-18-T replacement cartridges; Supelco Inc., Bellefonte, USA) at a constant flow rate of 1 ml min^−1^. The mobile phases were [i] buffer A [0.1 M KH_2_PO_4_/K_2_HPO_4_, with 4 mM tetrabutylammonium sulfate and 0.5% (v/v) CH_3_OH, pH = 6.0] and [ii] solvent B [70% (v/v) buffer A and 30% (v/v) CH_3_OH, pH = 7.2]. The following gradient program was implemented to separate the nucleotides in the samples: 100% buffer A from 0 min to 3.5 min, increase to 100% solvent B until 43.5 min, remaining at 100% solvent B until 51 min, decrease to 100% buffer A until 56 min, and remaining at 100% buffer A until 66 min.

The adenylate energy charge (AEC) is a quantitative measure of the relative saturation of high-energy phospho-anhydride bonds available in the adenylate pool of the cell [34,59], and can be expressed according to the formula:

$$ \mathrm{A}\mathrm{E}\mathrm{C}=\left(\left[\mathrm{A}\mathrm{T}\mathrm{P}\right]+0.5\xi \left[\mathrm{A}\mathrm{D}\mathrm{P}\right]\right)/\left(\left[\mathrm{A}\mathrm{T}\mathrm{P}\right]+\left[\mathrm{A}\mathrm{D}\mathrm{P}\right]+\left[\mathrm{A}\mathrm{M}\mathrm{P}\right]\right) $$

The AEC values were derived from the experimental measurements of each adenine nucleotide in the samples. The amount of ATP available per unit of biomass (*Y*_ATP/X_, in μmol ATP g_CDW_^−1^) was also calculated.

##### Calculation of maintenance demands

Growth-decoupled maintenance demands on glucose (*m*_S_, in g_glucose_ g_CDW_^−1^ h^−1^) were calculated by following the Pirt's equation [60]:

$$ {q}_s={m}_s+\upmu /{Y_{\mathrm{X}/\mathrm{S}}}^{true} $$

where *q*_S_ is the specific rate of glucose consumption (in g_glucose_ g_CDW_^−1^ h^−1^), μ is the specific growth rate (in h^−1^), and *Y*_X/S_^*true*^ is the true yield of biomass on glucose (in g_CDW_ g_glucose_^−1^).

A linear regression was used to calculate *m*_S_ values through a weighted least-squares regression. This method allows to take into account the variance of each data point individually, instead of assuming a constant variance. Weighted least-squares regression minimizes the error estimate (*s*) according to the following equation:

$$ s={\displaystyle {\sum}_i{\upomega}_{\mathrm{i}}}\times {\left({y}_{\mathrm{i}}-{\widehat{y}}_{\mathrm{i}}\right)}^2 $$

where ω_i_ is the *i*-th weight, and *y*_i_ and *ŷ*_i_ are the measured data points and the data points derived from regression, respectively. The weights determine how much each value influences the final parameter estimate [61]. Therefore, the fit is less influenced by data points of higher variance (σ_i_^2^) than sampling points with lower variance. The weights are calculated using the following equation:

$$ {\upomega}_{\mathrm{i}}=1/{\upsigma}_{\mathrm{i}}^2 $$

### Statistical analysis

The reported experiments were independently repeated at least twice (as indicated in the text), and, unless indicated otherwise, the mean value of the corresponding parameter ± standard deviation is presented. All continuous cultivations were carried out in independent biological triplicates, and each sample was additionally taken in technical triplicates. Differences in results were evaluated *via* a two-tailed Student's *t*-test defining a *P*-value < 0.05 as significant.

## Additional file

Additional file 1: Figure S1. Physiological characterization of (A) *P. putida* KT2440, (B) *P*. putida EM329, and (C) *P. putida* EM383 in glucose-limited chemostat cultures at different dilution rates (*D*). Figure S2. Carbon balance of glucose-limited chemostat cultures of *P. putida* KT2440, *P. putida* EM329, and *P. putida* EM383. Figure S3. Propidium iodide (PI) exclusion to estimate cell viability in *P. putida* KT2440, *P. putida* EM329, and *P. putida* EM383 with the empty and the recombinant plasmid. Figure S4. Physiological characterization in bioreactor batch cultivations of the different strains carrying plasmids. Figure S5. Physiological characterization in bioreactor batch cultivations of the different strains carrying plasmids.
