# Supplementary material for: Genome reduction boosts heterologous gene expression in Pseudomonas putida
Source: Microb Cell Fact. 2015 Feb 21;14:23. doi: 10.1186/s12934-015-0207-7 (PMC4352270; doi:10.1186/s12934-015-0207-7)
Supplement: Additional file 1: Figure S1. — Physiological characterization of (A) P. putida KT2440, (B) P. putida EM329, and (C) P. putida EM383 in glucose-limited chemostat cultures at different dilution rates (D). Figure S2. Carbon balance of glucose-limited chemostat cultures of P. putida KT2440, P. putida EM329, and P. putida EM383. Figure S3. Propidium iodide (PI) exclusion to estimate cell viability in P. putida KT2440, P. putida EM329, and P. putida EM383 with the empty and the recombinant plasmid. Figure S4. Physiological characterization in bioreactor batch cultivations of the different strains carrying plasmids. Figure S5. Physiological characterization in bioreactor batch cultivations of the different strains carrying plasmids. [file 12934_2015_207_MOESM1_ESM.pdf]

ADDITIONAL FILE

# Genome reduction boosts heterologous gene expression in *Pseudomonas putida*

by

Sarah Lieder, Pablo I. Nikel, Víctor de Lorenzo, and Ralf Takors

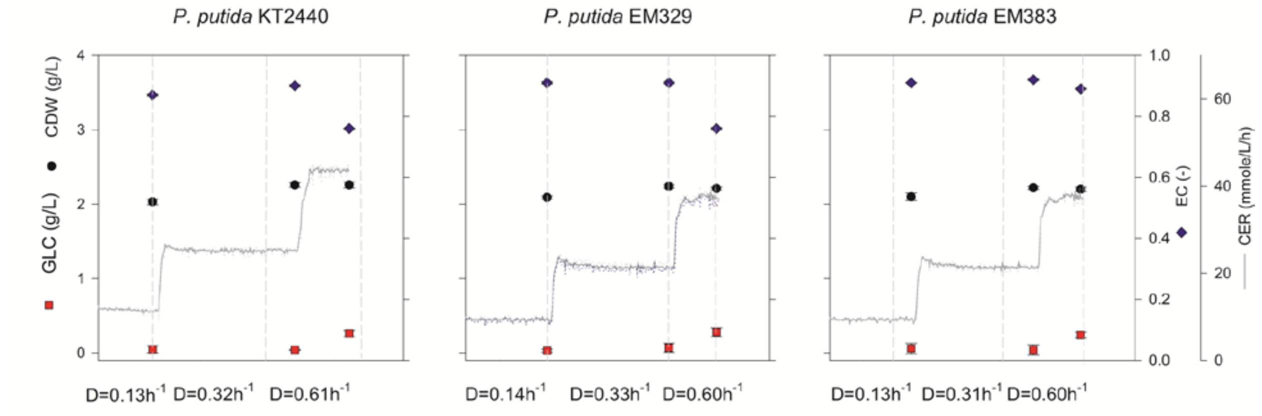


FIG. S1. Physiological characterization of (A) *P. putida* KT2440, (B) *P*. putida EM329, and (C) *P*. *putida* EM383 in glucose-limited chemostat cultures at different dilution rates (*D*). Each cultivation was performed in biological triplicates. *D* was increased step-wise from *D* = 0.1 to 0.3 and 0.6 $h^{-1}$ after five residence times at each *D* value when a steady state was achieved. Steady states were monitored by the stable carbon emission rate (CER, black line) and stable optical density measurements (data not shown). Cell dry weight (CDW, black dots), residual glucose concentration (GLC, red squares), and the adenylate energy charge (EC, blue diamonds) were measured at steady state conditions after 5 residence times of one specific dilution rate. Error bars represent standard deviations of the biological triplicates.

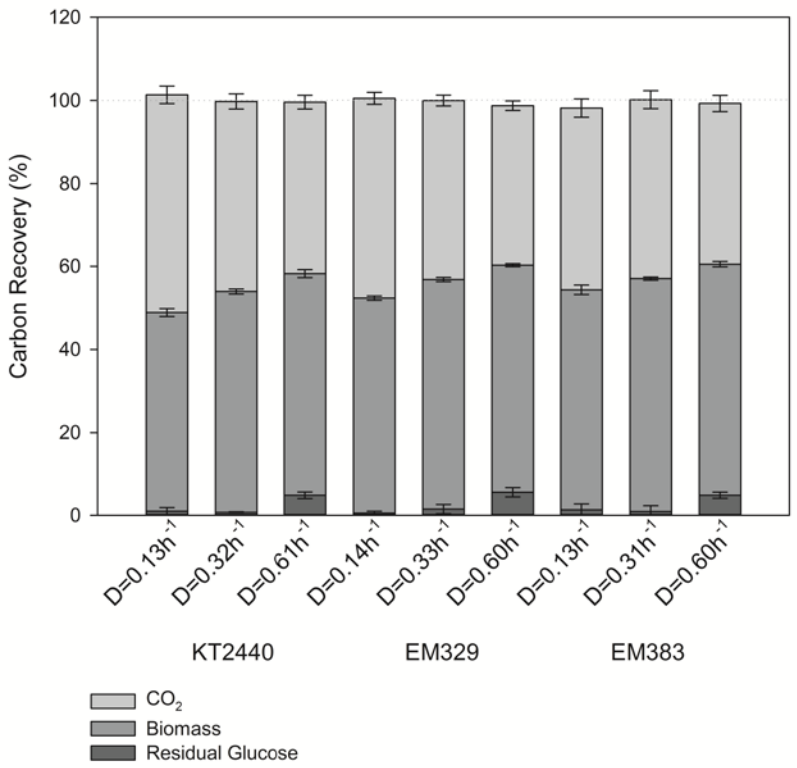


**FIG. S2.** Carbon balance of glucose-limited chemostat cultures of *P. putida* KT2440, *P. putida* EM329, and *P. putida* EM383. The carbon provided by glucose served as the 100% carbon input into the cultivation. Carbon recovery (%) was calculated considering residual glucose concentrations (dark grey), cell dry weight concentrations (grey), and $CO_2$ emission (light grey). Error bars represent standard deviations of the biological triplicates.

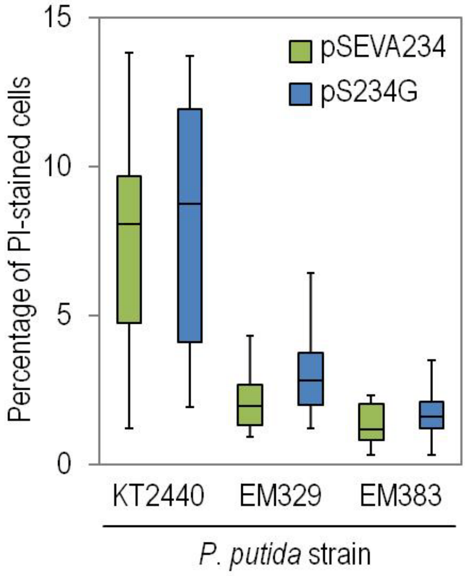


FIG. S3. Propidium iodide (PI) exclusion was used to estimate cell viability in *P. putida* KT2440, *P. putida* EM329, and *P. putida* EM383 with the empty and the recombinant plasmid. Appropriate dilutions of cell suspensions grown on M12 minimal medium with 10 g $l^{-1}$ glucose were stained with PI and the percentage of PI-positive cells was determined by flow cytometry as detailed in the Material and Methods section. Box plots represent the median value and the 1st and 3rd quartiles of the geometric mean values of quadruplicate determinations from three independent cultures, and the asterisks identify significant differences at the $P < 0.05$ level as assessed with the Mann-Whitney *U* test.

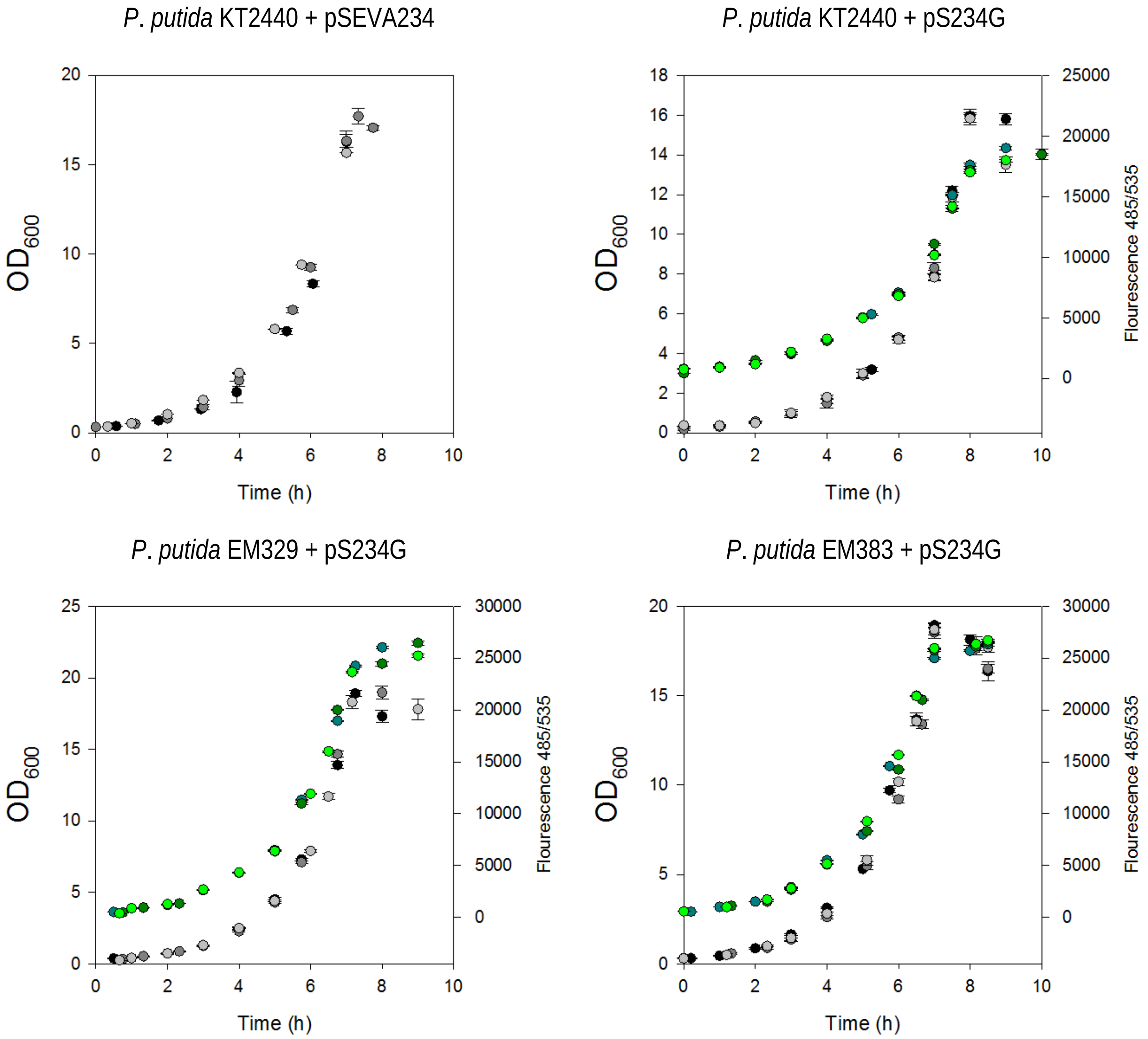


FIG. S4. Physiological characterization in bioreactor batch cultivations of the different strains carrying plasmids. Batch cultivations were carried out with glucose as sole carbon source in a working volume of 1.5 liter in biological triplicates. The time course of the cultivations was monitored via biomass concentration (CDW; black, grey, and light grey dots) and in the case of the strains carrying GFP on the plasmid (pSEVA234G), the GFP fluorescence [GFP, measured in arbitrary flourescence units (A.F.U.), dark green, green, and light green dots] was measured throughout the cultivation.

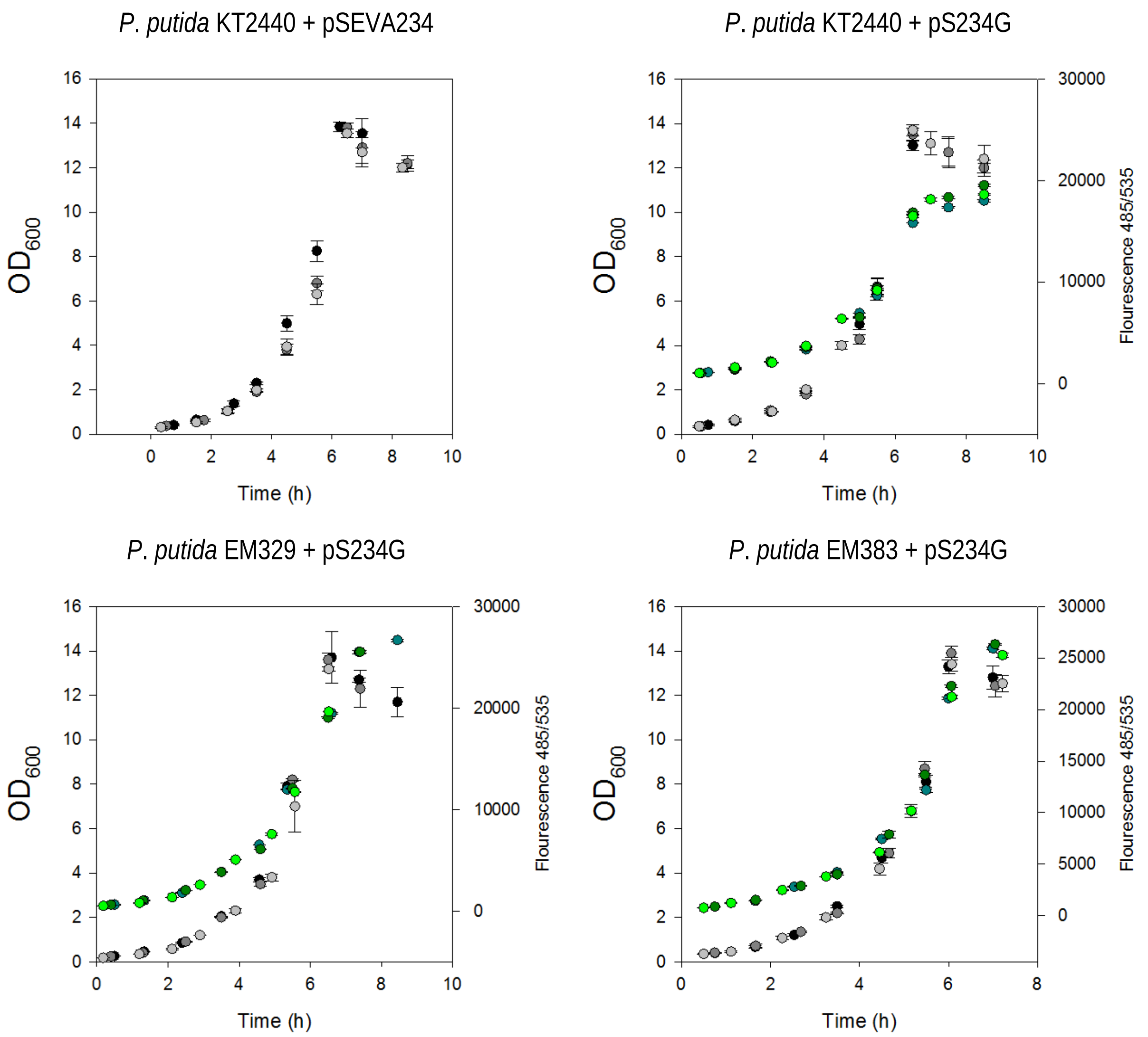


FIG. S5. Physiological characterization in bioreactor batch cultivations of the different strains carrying plasmids. Batch cultivations were carried out with citrate as sole carbon source in a working volume of 1.5 liter in biological triplicates. The time course of the cultivations was monitored via biomass concentration (CDW; black, grey, and light grey dots) and in case of the strains carrying GFP on the plasmid (pSEVA234G), the GFP fluorescence [GFP, measured in arbitrary flourescence units (A.F.U.), dark green, green, and light green dots] was measured throughout the cultivation.
